# Supplementary material for: High Concentration or Combined Treatment of Antisense Oligonucleotides for Spinal Muscular Atrophy Perturbed SMN2 Splicing in Patient Fibroblasts
Source: Genes (Basel). 2022 Apr 13;13(4):685. doi: 10.3390/genes13040685 (PMC9027857; doi:10.3390/genes13040685)
Supplement: Supplementary file 1 [file genes-13-00685-s001.zip › 20220413 Supplementary tables.pdf]

Supplementary Table S1. Dose effects of ASOs on *SMN2* splicing pattern: densitometry data

		dH2O	ASO-NUS			ASO-EX8			ASO-SSJ		
			50	100	200	50	100	200	50	100	200
Ex6/Ex7/In7/Ex8	Mean	0.00	0.00	0.00	0.00	trace	trace	12350.00	trace	trace	trace
(Intron 7-retention)	[SD]	0.00	0.00	0.00	0.00	-	-	401.43	-	-	-
Ex6/Ex7a/Ex7/Ex8	Mean	0.00	0.00	0.00	26335.00	0.00	0.00	20624.00	0.00	0.00	12747.45
(Ex7a-inclusion)	[SD]	0.00	0.00	0.00	224.17	0.00	0.00	478.21	0.00	0.00	29.17
Ex6/Ex7/Ex8	Mean	10430.50	26590.12	32882.02	32255.23	25970.89	27130.64	14797.72	30965.13	12709.60	10044.55
	[SD]	260.98	7.53	98.96	60.13	42.41	30.32	132.46	101.11	30.25	53.71
Ex6/Ex8	Mean	25130.68	21051.24	4807.86	13677.30	12789.16	19630.76	19502.62	14792.37	26341.05	21524.69
	[SD]	1255.60	364.37	332.23	407.57	25.36	237.55	323.41	242.47	331.85	383.49
GAPDH	Mean	33722.02	31291.89	30532.87	30161.77	31147.08	30150.06	28300.53	32590.50	29673.50	26159.22
	[SD]	86.89	106.50	117.64	47.34	93.29	64.39	227.10	218.33	187.69	50.98

Supplementary Table S2. Dose effects of ASOs on *SMN2* splicing pattern: densitometry data (*SMN2* transcript level/*GAPDH* level)

		dH2O	ASO-NUS			ASO-EX8			ASO-SSJ		
			50	100	200	50	100	200	50	100	200
Ex6/Ex7/In7/Ex8	Mean	0.00	0.00	0.00	0.00	trace	trace	0.44	trace	trace	trace
(Intron 7-retention)	[SD]	0.00	0.00	0.00	0.00	-	-	0.01	-	-	-
Ex6/Ex7a/Ex7/Ex8	Mean	0.00	0.00	0.00	0.87	0.00	0.00	0.73	0.00	0.00	0.49
(Ex7a-inclusion)	[SD]	0.00	0.00	0.00	0.01	0.00	0.00	0.01	0.00	0.00	0.00
Ex6/Ex7/Ex8	Mean	0.31	0.85	1.08	1.07	0.83	0.90	0.52	0.95	0.43	0.38
	[SD]	0.01	0.00	0.01	0.00	0.00	0.00	0.01	0.01	0.00	0.00
Ex6/Ex8	Mean	0.75	0.67	0.16	0.45	0.41	0.65	0.69	0.45	0.89	0.82
	[SD]	0.04	0.01	0.01	0.01	0.00	0.01	0.01	0.00	0.01	0.01

Supplementary Table S3. Dose effects of ASOs on *SMN2* splicing pattern: Full-length/delta-7 ratio

		dH2O	ASO-NUS			ASO-EX8			ASO-SSJ		
			50	100	200	50	100	200	50	100	200
Full-length / delta-7 ratio	Mean	0.42	1.26	6.87	2.36	2.03	1.38	0.76	2.09	0.48	0.47
	[SD]	0.02	0.02	0.44	0.07	0.00	0.02	0.02	0.04	0.01	0.01

Supplementary Table S4. Combination effects of ASOs on *SMN2* splicing pattern: densitometry data

		dH2O	ASO-NUS	ASO-EX8	ASO-SSJ	ASO-NUS / ASO-EX8	ASO-NUS / ASO-SSJ	ASO-EX8 / ASO-SSJ
Ex6/Ex7/In7/Ex8	Mean	0.00	0.00	trace	trace	trace	trace	39694.21
(Intron 7-retention)	[SD]	0.00	0.00	-	-	-	-	239.13
Ex6/Ex7a/Ex7/Ex8	Mean	0.00	0.00	0.00	0.00	17184.59	25994.98	trace
(Ex7a-inclusion)	[SD]	0.00	0.00	0.00	0.00	257.07	190.06	-
Ex6/Ex7/Ex8	Mean	25818.63	47905.91	30041.50	28886.11	35663.21	30998.04	13182.53
	[SD]	68.02	119.64	214.47	279.53	56.58	24.50	96.63
Ex6/Ex8	Mean	30385.77	1781.11	9917.55	8538.18	4658.79	6486.39	14161.24
	[SD]	153.49	101.65	187.12	454.66	164.04	109.694616	313.46
GAPDH	Mean	48984.24	43108.71	37577.08	45054.82	39015.97	37986.74	35996.07
	[SD]	316.53	111.34	130.38	126.37	259.85	234.96	136.35

Supplementary Table S5. Combination effects of ASOs on *SMN2* splicing pattern: densitometry data (adjusted to GAPDH level)

		dH2O	ASO-NUS	ASO-EX8	ASO-SSJ	ASO-NUS / ASO-EX8	ASO-NUS / ASO-SSJ	ASO-EX8 / ASO-SSJ
Ex6/Ex7/In7/Ex8	Mean	0.00	0.00	trace	trace	trace	trace	1.10
(Intron 7-retention)	[SD]	0.00	0.00	-	-	-	-	0.01
Ex6/Ex7a/Ex7/Ex8	Mean	0.00	0.00	0.00	0.00	0.44	0.68	trace
(Ex7a-inclusion)	[SD]	0.00	0.00	0.00	0.00	0.01	0.01	-
Ex6/Ex7/Ex8	Mean	0.53	1.11	0.80	0.64	0.91	0.82	0.37
	[SD]	0.00	0.01	0.00	0.01	0.01	0.00	0.00
Ex6/Ex8	Mean	0.62	0.04	0.26	0.19	0.12	0.17	0.39
	[SD]	0.01	0.00	0.01	0.01	0.00	0.00	0.01

Supplementary Table S6. Combination effects of ASOs on *SMN2* splicing pattern: Full-length/delta-7 ratio

		dH2O	ASO-NUS	ASO-EX8	ASO-SSJ	ASO-NUS / ASO-EX8	ASO-NUS / ASO-SSJ	ASO-EX8 / ASO-SSJ
Full-length / delta-7 ratio	Mean	0.23	7.29	0.82	0.92	2.07	1.29	0.25
	[SD]	0.00	0.42	0.02	0.06	0.07	0.02	0.01
