# Supplementary material for: High Concentration or Combined Treatment of Antisense Oligonucleotides for Spinal Muscular Atrophy Perturbed SMN2 Splicing in Patient Fibroblasts
Source: Genes (Basel). 2022 Apr 13;13(4):685. doi: 10.3390/genes13040685 (PMC9027857; doi:10.3390/genes13040685)
Supplement: Supplementary file 1 [file genes-13-00685-s001.zip › genes--supplementary figure.pdf]

Supplementary Figure S1.

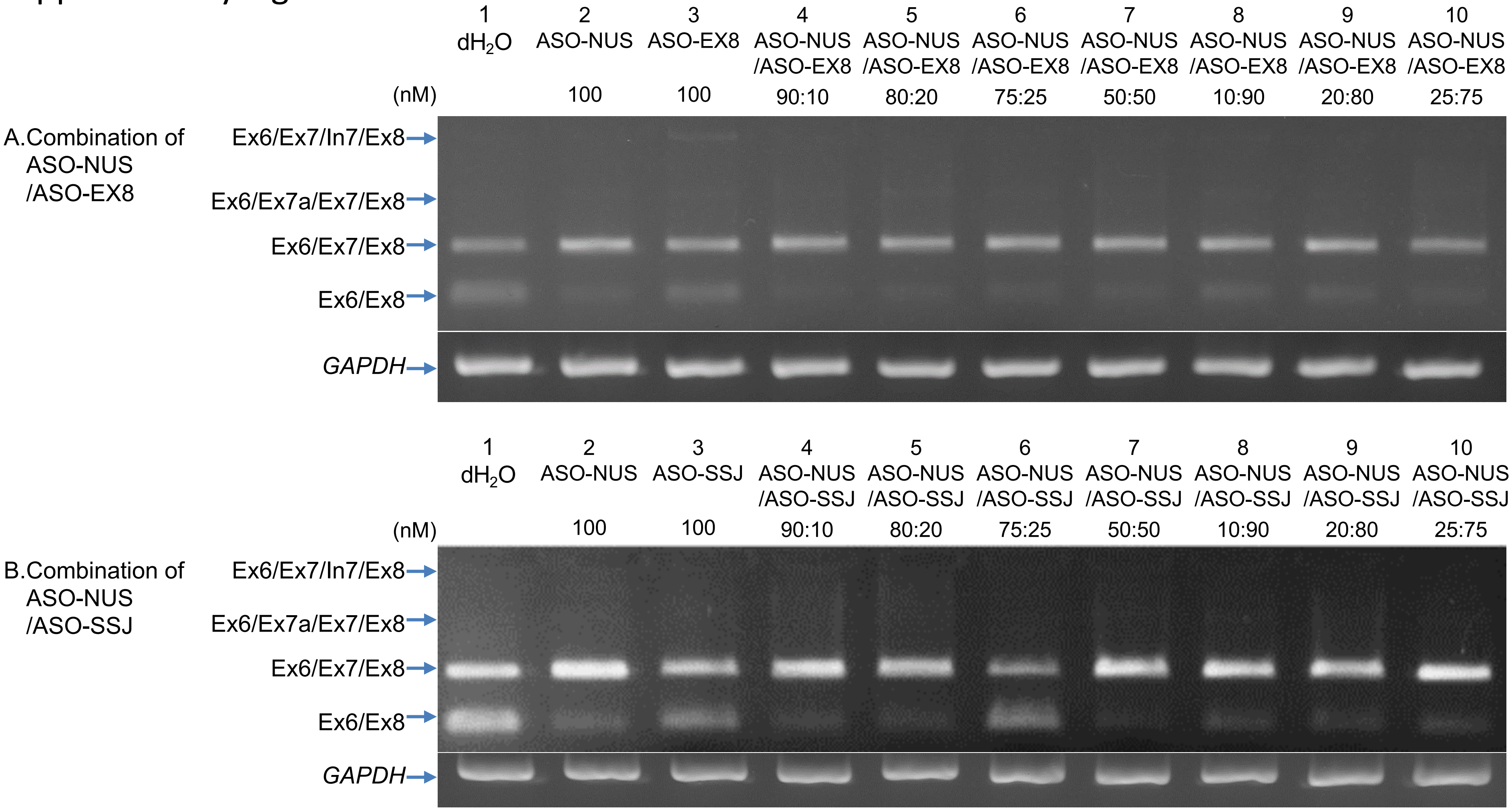

Supplementary Figure S1. Transcript analysis of a total dose of 100 nM with various combination ratios from (A) ASO-NUS/ASO-EX8 and (B) ASO-NUS/ASO-SSJ experiments. The types and concentrations of ASOs used in this study are indicated above the gel pictures. The arrows indicate the transcript product of *SMN2* (upper panel) or *GAPDH* (lower panel).

The ASO combinations in this experiment corrected the *SMN2* exon 7 splicing to some degree. However, it should be noted here that the cryptic exon activation was not clearly observed in all groups with a total dose of 100 nM. As shown in Figures 4A and 4C of the text, the combination of ASO-NUS/ASO-EX8 (100 nM each) or ASO-NUS/ASO-SSJ (100 nM each) produced a high level of the exon 7a-containing transcript (Ex6/Ex7a/Ex7/Ex8), indicating cryptic exon activation occurred at a total dose of 200 nM. Taken together, the total dose might be more critical than combination ratios of ASOs in the cryptic exon activation.
